# Kinetic and radiomic features on DCE-MRI as a predictor for axillary lymph node metastasis burden in T1 and T2 stage breast cancer

**DOI:** 10.3389/fonc.2025.1700248

**Published:** 2026-01-07

**Authors:** Yuanyuan Liu, Chunhua Wang, Ruirui Meng, Hongbing Luo, Xiaoyu Chen, Shaoyu Wang, Jing Ren, Peng Zhou, Xin Zhang

**Affiliations:** 1 Department of Radiology, Sichuan Clinical Research Center for Cancer, Sichuan Cancer Hospital & Institute, Sichuan Cancer Center, Affiliated Cancer Hospital of University of Electronic Science and Technology of China, Chengdu, China; 2 MR Scientific Marketing, Siemens Healthineers, Shanghai, China; 3 Department of Breast Surgery, Sichuan Clinical Research Center for Cancer, Sichuan Cancer Hospital & Institute, Sichuan Cancer Center, Affiliated Cancer Hospital of University of Electronic Science and Technology of China, Chengdu, China

**Keywords:** breast cancer, kinetics, lymph node, magnetic resonance imaging, metastasis, radiomics

## Abstract

**Background:**

Magnetic resonance imaging (MRI) is increasingly used to evaluate axillary lymph node (ALN) status in breast cancer. However, the correlation between MRI features of the primary tumor and the ALN metastasis (ALNM) burden remains poorly understood. This study aimed to develop a non-invasive MRI-based model to preoperatively distinguish between low (≤2 nodes) and high (>2 nodes) ALNM burden in T1 and T2 stage breast cancer.

**Methods:**

This retrospective single-center study included 185 patients, categorized by ALNM burden [≤ 2 nodes (n = 149) or >2 nodes (n = 36)]. The kinetic and radiomic features were extracted from the segmented whole tumor on dynamic contrast-enhanced MRI (DCE-MRI). A forward-stepwise feature selection method was employed based on the ANOVA F-score from the training cohort. Features were added according to F-values and logistical regression model was built iteratively. The final model, trained on the entire training set, was evaluated on the independent test cohort.

**Results:**

The model incorporated five kinetic and three radiomic features, demonstrating moderate predictive performance. The model achieved an area under the receiver operating characteristic curve (AUC) of 0.705 in the test cohort. It showed a sensitivity of 72.7% and a specificity of 77.8%. The negative predictive value (NPV) was 92.1%.

**Conclusion:**

The kinetic and radiomic features from DCE-MRI showed potential for predicting ALNM burden (≤2 or > 2 nodes) in T1 and T2 stage breast cancer. The high NPV particularly supported their utility as a non-invasive tool to identify candidates for less invasive axillary procedures.

## 1 Introduction

Breast cancer remains one of the most common malignant tumors among women worldwide (1, 2). Axillary lymph node metastasis (ALNM) is a key determinant of overall recurrence and survival in breast cancer patients (3). Since the ACOSOG Z0011 trial (4), there has been a significant change in the management of axillary lymph nodes (ALN). This trial demonstrated that ALN dissection (ALND) could be omitted in T1 or T2 stage breast cancer patients with two or fewer metastatic sentinel lymph nodes (SLNs). A non-invasive method to preoperatively determine ALNM burden (≤2 or > 2 nodes) could facilitate more individualized treatment plans, such as guiding the choice between SLN biopsy (SLNB) alone and direct ALND, or identifying candidates for neoadjuvant chemotherapy.

Magnetic resonance imaging (MRI) is playing an increasingly important role in evaluating both the primary tumor and ALN status in breast cancer (5). Recently, radiomics has gained considerable attention for its ability to extract quantitative imaging features that may reveal invisible disease characteristics and tumor heterogeneity (6). Many previous studies have focused on MRI-based radiomic features derived from the primary breast tumor to predict ALN status. Evidence suggested that radiomic features extracted from MRI could serve as a non-invasive quantitative method to predict ALNM or SLN metastases in breast cancer (7–16). When combined with or without clinicopathological variables, radiomic models reported area under the receiver operating characteristic (ROC) curve (AUC) values varying from 0.60 to 0.91 for predicting ALNM or SLN metastases (7–16). Few studies have focused on MRI radiomics in evaluating ALNM burden. In one study, a radiomic signature achieved an AUC of 0.79 in distinguishing the number of metastatic ALNs (≤2 or > 2 nodes) (7).

The kinetic features of the primary tumor from dynamic contrast-enhanced MRI (DCE-MRI) have also been investigated for their relationship with ALNM. Several studies (17–20) have reported significant correlations of ALNM with kinetic parameters, such as the skewness of K_ep_, K^trans^ value, enhancement maximum slop, time to peak (TTP) and initial peak enhancement (PE) (all *p* < 0.05). Furthermore, one study (21) found that combined metrics—PE multiplied by tumor volume (PE × volume) and total percent persistent enhancement—could predict the presence of four or more metastatic ALNs, with a combined model achieving an AUC of 0.79. Conversely, Choi EJ et al. (22) concluded that semi-quantitative kinetic parameters (e.g., PE percentage and TTP) were not useful for predicting SLN metastases, as did Rahbar H et al. (23). Our previous study (24) in a relatively small sample found no association between ALNM and quantitative kinetic parameters, including their histogram and texture features. The integration of kinetic parameters with radiomics has been explored in very few studies. To date, the relationship between quantitative kinetic features and the specific number of metastatic ALNs (≤2 or >2 nodes) remains uninvestigated.

The primary aim of this study was to validate the association between quantitative kinetic features and ALNM in a larger sample size. The second was to develop and validate a non-invasive MRI-based prediction model that combined both kinetic and radiomic features from the primary tumor, aiming to preoperatively distinguish between low (≤2 nodes) and high (>2 nodes) ALNM burden.

## 2 Methods

### 2.1 Subjects

The study received approval from our hospital’s institutional review board (SCCHEC2015029), and all enrolled patients provided written informed consent. Consecutive patients at Sichuan Cancer Hospital were retrospectively recruited during the period from 01/08/2015 to 31/03/2018. Patients were included if they met the following criteria: 1) pathologically confirmed primary breast cancer; 2) T1 and T2 stage breast cancer, as well as ductal carcinoma *in situ* (DCIS); 3) DCE-MRI examination before biopsy or surgery; 4) DCE-MRI examination on the same 3.0-T MRI scanner in our department; and 5) pathological diagnosis for ALN by SLNB and/or ALND. Patients were excluded based on the following: 1) no surgical intervention and thus no pathological ALN diagnosis, or receiving neoadjuvant chemotherapy (NAC); 2) advanced breast cancer; 3) receiving chemotherapy or radiotherapy before the MRI examination; 4) interval between MRI scan and surgery exceeding 1 week; and 5) unavailable quantitative parameters due to data processing failures or unsatisfactory imaging quality. The following clinicopathological parameters were collected from medical records: age, menopausal status, tumor size, axillary surgical approach, histopathological type, estrogen receptor (ER), progesterone receptor (PR), human epidermal growth factor receptor 2 (HER2) status, and the Ki-67 proliferation index.

### 2.2 MRI acquisition

All patients underwent MRI scanning in the prone position using the same 3.0-T MRI device (Siemens Healthcare, Erlangen, Germany) equipped with a dedicated 16-channel breast array coil. The detailed imaging protocol is provided in **Supplementary Table S1**. The dynamic scanning involved 26 consecutive phases employing the CAIPIRINHA-Dixon-Twist-Vibe sequence.

### 2.3 Post-processing of MRI images

#### 2.3.1 Whole tumor segmentation

**Figure 1:**
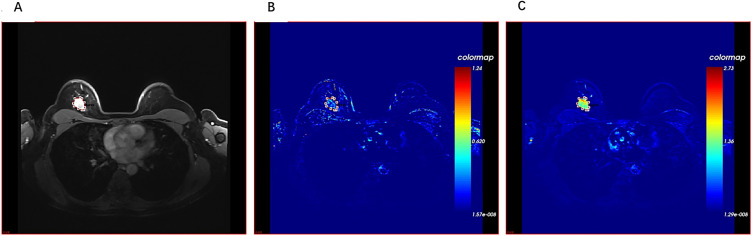
A 51-year-old woman diagnosed with invasive carcinoma of no special type (NST) with negative axillary lymph nodes. **(A)** Tumor region of interest (ROI) outlined on the early-phase DCE-MRI images. **(B)** K_ep_ color-coded map. **(C)** K^trans^ color-coded map. DCE-MRI, dynamic contrast-enhanced magnetic resonance imaging.

Raw DCE-MRI data were initially processed using dedicated post-processing software, Omni-Kinetics (GE Healthcare, Milwaukee, WI, USA). The reference region model (25) was employed using the pectoralis major muscle as the reference region. Two radiologists (with more than 8 years of experience), blinded to the pathological information of ALN, manually delineated the entire tumor slice by slice on the early post-contrast DCE-MRI images (**Figure 1**). When a tumor presented with spiculations, they were included in the region of interest (ROI) delineation; however, the peritumoral tissue was excluded. For patients with multiple lesions, the largest tumor was selected for analysis. All ROIs were subsequently reviewed and confirmed by a third radiologist with 15 years of experience. The final ROIs were merged into a single three-dimensional (3D) volume of interest (VOI). As noted in our previous study (11), to ensure the reproducibility of the extracted kinetic and radiomic features, images from 20 randomly selected patients were used for intra- and inter-observer agreement analyses. One radiologist performed the VOI segmentation twice at 1-month intervals to assess intra-observer consistency, while a second radiologist independently segmented the same set to evaluate inter-observer agreement. The reliability was calculated using the intraclass correlation coefficient (ICC). Features with ICC greater than 0.75 were retained for further analysis.

#### 2.3.2 Kinetic feature extraction

The software automatically generated a comprehensive set of quantitative and semi-quantitative kinetic parameters from the VOI, as illustrated in **Figure 1**. These parameters included the following: 1) K^trans^ (min^−1^), the volume-transfer constant, indicative of vascular permeability and perfusion; 2) K_ep_ (min^−1^), the washout-rate constant, reflecting contrast agent reflux back to the vessels; 3) volume fraction of plasma (Vp); 4) voxel value; 5) TTP; 6) blood flow (BF); 7) blood volume (BV); 8) maximum concentration (MAX Conc); 9) MAX Slope; 10) AUC; and 11) mean transit time (MTT). For each of these parameters, the software also provided a range of statistical metrics, including the maximum, minimum, median, mean, Std, and area, as well as the 10%, 25%, 50%, 75%, and 90% values.

#### 2.3.3 Radiomic feature extraction

The radiomic features of the tumors were automatically extracted from the early post-contrast DCE-MRI images using the defined VOI. The software generated a total of 77 features, categorized as follows: morphology metrics (n = 9), first-order statistical features (n = 29), gray-level co-occurrence matrix (GLCM) features (n = 13), Haralick features (n = 10), and run-length matrix (RLM) features (n = 16).

#### 2.3.4 Feature selection and model building

**Figure 2:**
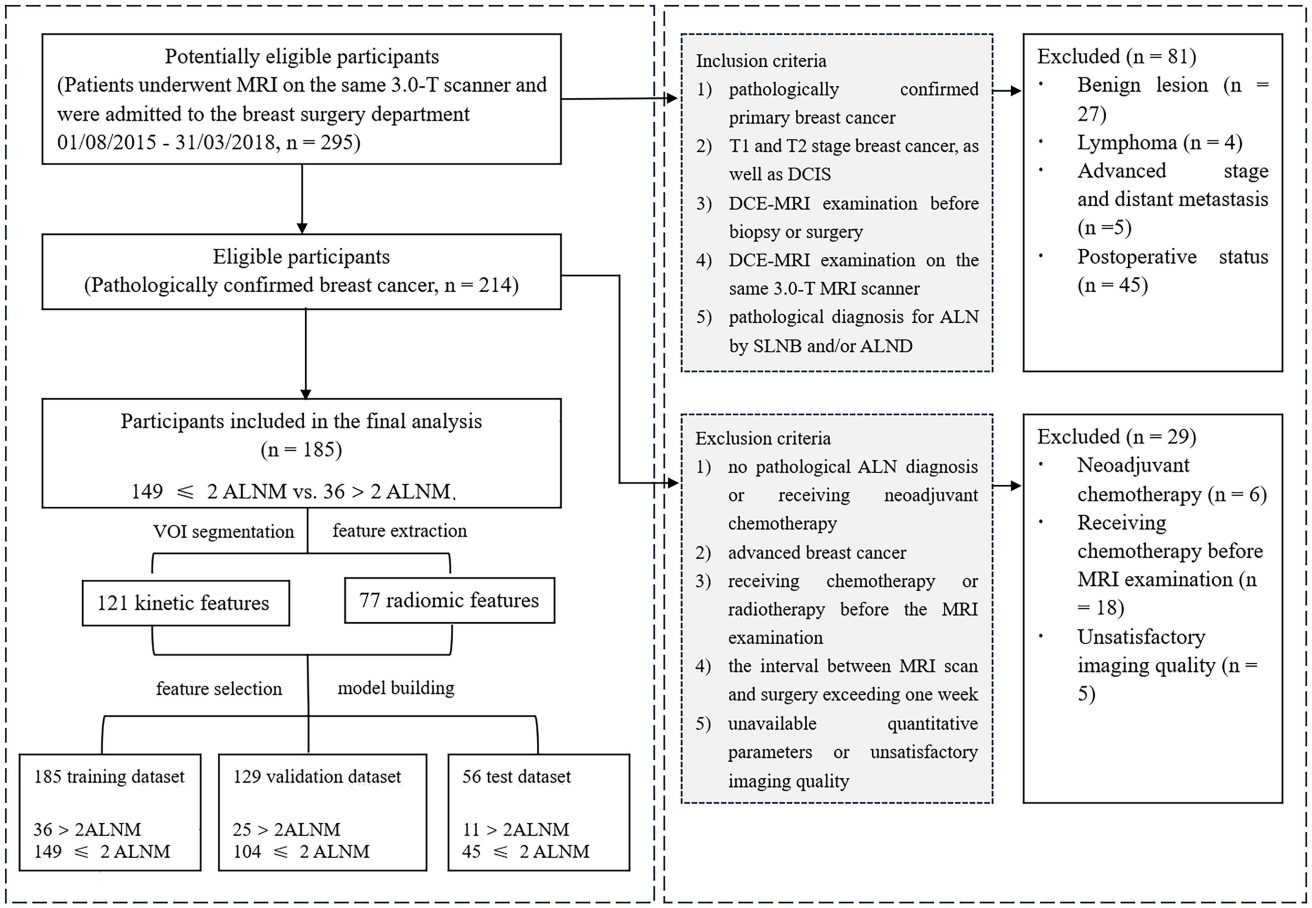
The study workflow. MRI, magnetic resonance imaging; ALNM, axillary lymph node metastasis; VOI, volume of interest; DCIS, ductal carcinoma *in situ*; DCE-MRI, dynamic contrast-enhanced MRI; ALN, axillary lymph node; SLNB, sentinel lymph node biopsy; ALND, axillary lymph node dissection.

The dataset comprised 185 cases, and we used a random split method to divide the cohort into training (70%) and test (30%) sets, ensuring that the test cohort was completely independent from the training process. The training set consisted of 129 cases (25, >2 metastatic nodes; 104, ≤2 metastatic nodes), while the independent test set included 56 cases (11, >2 metastatic nodes; 45, ≤2 metastatic nodes). Prior to model development, the feature matrix underwent normalization. Specifically, we scaled each feature vector by its L2 norm, effectively transforming it into a unit vector. To address the high dimensionality of the feature space, we performed a feature reduction step. We computed the cosine similarity for all feature pairs; if the cosine value between any two features exceeded 0.86, we removed one of them. This procedure effectively reduced the feature space dimension and ensured greater independence among the remaining features. We used a forward-stepwise feature selection method to select the set of features. We implemented analysis of variance (ANOVA) on the training cohort, and we estimated the F-value for each feature. Then, we added each feature according to the F-value and built a logistic regression model iteratively. For each set of features, we used 10-fold cross-validation to evaluate the performance and to determine the selected features. Then, we built the model based on all training cases and evaluated the model on the independent test cohort. To prevent overfitting and ensure a rigorous evaluation of the model’s generalizability, we strictly confined all feature selection and model-building steps to the training cohort. We then evaluated the final model on the completely independent test cohort, the results of which were reported herein. **Figure 2** shows the study workflow.

### 2.4 Statistical analysis

Model performance was assessed using ROC curve analysis, with the AUC calculated for quantification evaluation. Additional metrics—including sensitivity, specificity, positive predictive value (PPV), and negative predictive value (NPV)—were computed at the optimal cutoff threshold determined by maximizing Youden’s index. The estimation was also boosted 1,000 times, and a paired t-test was applied to give the 95% confidence interval. The entire analytical workflow was executed in Python 3.7.6, utilizing the FeAture Explorer Pro (FAE, v0.6.4, https://github.com/salan668/FAE).

Continuous variables with normal and non-normal distributions were compared using Student’s t-test and the Mann–Whitney U test and were presented as mean ± standard deviation and median [interquartile range (IQR)], respectively. Categorical variables were compared using the chi-square test or Fisher’s exact test to assess for differences in clinical and pathological factors between the ALNM groups (>2 *vs*. ≤2 nodes). Statistical analyses were performed using the R software (v4.2.3, http://www.r-project.org). A two-tailed p value <0.05 was considered statistically significant.

## 3 Results

There were 149 patients with ALN metastases of less than or equal to two nodes and 36 patients with ALN metastases of more than two nodes. The clinicopathological characteristics of patients are listed in **Table 1**. The clinical and pathological characteristics—including age, menopause status, tumor size, histopathological type, ER/PR positivity rate, HER2 positivity rate, and molecular type—did not differ significantly between the ALNM groups (>2 *vs*. ≤2 nodes). A significantly higher proportion of T2 stage tumors and Ki-67 positivity was found in the group with ALNM > 2 nodes, which was consistent with clinical observations. A higher SLNB rate was observed in the group with ALNM ≤ 2 nodes.

**Table 1:** Clinical and pathological characteristics.

Variables	ALNM ≤ 2 nodes (n = 149)	ALNM > 2 nodes (n = 36)	*p*-Value
Age (years), median (IQR)	47 (42, 56)	48 (43,53)	0.067
Menopause (n, %)			0.897
Yes	68 (45.6)	16 (44.4)	
No	81 (54.4)	20 (55.6)	
Tumor size (mm), median (IQR)	21 (16, 26)	23 (18, 28)	0.067
Tumor stage (n, %)			0.002
T1	55 (36.9)	5 (13.9)	
T2	80 (53.7)	31 (86.1)	
Tis	14 (9.4)	0	
Number of ALNM (n, %)			<0.001
0	103 (69.1)	0	
1–2	46 (30.9)	0	
>2	0	36 (100)	
Axillary management (n, %)			<0.001
SLNB	109 (73.2)	0	
ALND/SLNB + ALND	40 (26.8)	36 (100)	
Histopathological type (n, %)			0.131
NST	129 (86.6)	35 (97.2)	
ILC	2 (1.3)	1 (2.8)	
DCIS	14 (9.4)	0	
Other types	4 (2.7)	0	
Pathological parameters (n, %)			
ER positivity ^a^	119/149 (79.9)	26/36 (72.2)	0.367
PR positivity ^a^	105/149 (70.5)	21/36 (58.3)	0.169
HER2 positivity ^b^	39/137 (28.5) ^d^	10/36 (27.8)	0.407
Ki-67 positivity ^c^	104/147 (70.7) ^d^	33/36 (91.7)	0.009
Molecular subtype (n, %)^e^			0.081
Luminal A	30 (20.1)	2 (5.6)	
Luminal B	81 (54.4)	24 (66.7)	
HER2-enrich	11 (7.4)	4 (11.1)	
TNBC	15 (10.1)	6 (16.7)	

ALNM, axillary lymph node metastasis; SLNB, sentinel lymph node biopsy; ALND, axillary lymph node dissection; NST, no special type; ILC, invasive lobular carcinoma; DCIS, ductal carcinoma *in situ*; ER, estrogen receptor; PR, progesterone receptor; HER2, human epidermal growth factor receptor 2; TNBC, triple-negative breast cancer; IQR, interquartile range.

^a^ER and PR positivity was defined as ≥1% of tumor cells appearing immunostained.

^b^HER2 positivity was defined as hematoxylin–eosin (H&E) staining 3+ or H&E staining 2+ with positive fluorescence in *in situ* hybridization test.

^c^Ki-67 positivity was considered as ≥14% cells appearing immunostained.

^d^For patients with DCIS, HER2 testing was not performed in 12 cases, and Ki-67 testing was not performed in two cases.

^e^Molecular type could not be determined for 12 DCIS cases due to unavailable HER2 data.

**Figure 3:**
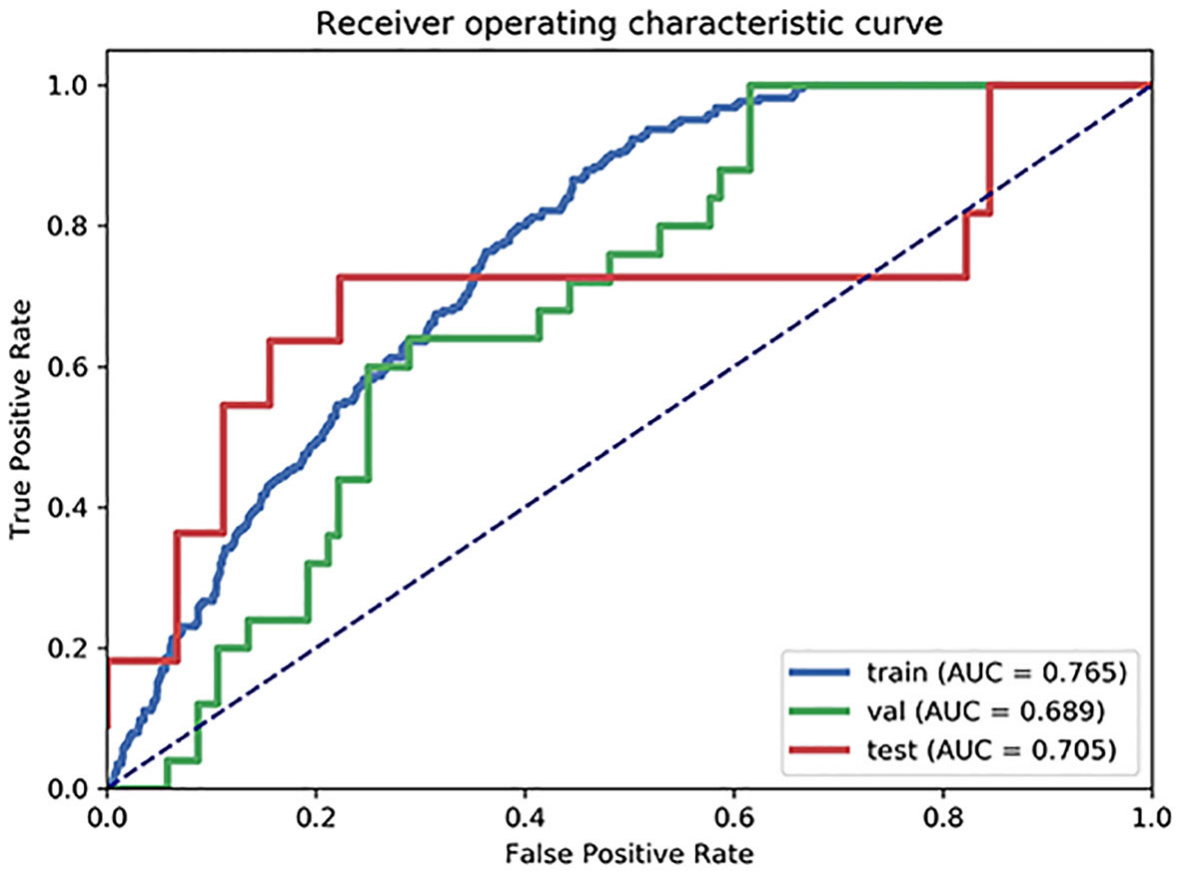
Receiver operating characteristic (ROC) curves of kinetic and radiomic feature model.

The final model, which incorporated five kinetic and three radiomic features, achieved the highest AUC. On the validation set, it yielded an AUC of 0.689, with a sensitivity of 68.0%, a specificity of 71.2%, a PPV of 36.2%, and a NPV of 90.2%. Performance on the test set was comparable, with an AUC of 0.705, a sensitivity of 72.7%, a specificity of 77.8%, a PPV of 44.4%, and a NPV of 92.1%. The diagnostic statistics and selected features are summarized in **Tables 2** and **3**, respectively. The corresponding ROC curves are shown in **Figure 3**.

**Table 2:** The performance of the kinetic and radiomic feature model in predicting ALNM burden (≤2 or >2 nodes).

Kinetic and radiomic feature model	Sensitivity (%)	Specificity (%)	PPV (%)	NPV (%)	Accuracy (%)	AUC
Training cohort (n = 185)	83.6	56.7	31.7	93.5	61.9	0.765
Validation cohort (n = 129)	68.0	71.2	36.2	90.2	70.5	0.689
Test cohort (n = 56)	72.7	77.8	44.4	92.1	76.8	0.705

ALNM, axillary lymph node metastasis; PPV, positive predictive value; NPV, negative predictive value; AUC, area under the curve.

**Table 3:** The selected features and their coefficients in the model.

The selected kinetic and radiomic features	Coefficient in model
Area of tumor (total number of voxels within the tumor)	0.176
10% of K^trans^	−0.520
50% of K^trans^	0.394
50% of K_ep_	−0.446
Mean TTP	−0.532
GlcmEnergy	−0.557
HaralickCorrelation	0.242
LowGreyLevelRunEmphasis	−0.386

TTP, time to peak.

After excluding DCIS cases, 135 patients had ≤2 metastatic ALNs, and 36 patients had >2 metastatic ALNs (clinicopathological characteristics in **Supplementary Table S2**). The final model on the test set, which incorporated three kinetic and three radiomic features, achieved an AUC of 0.714. However, the predictive performance showed a decrease accordingly, with a sensitivity of 63.6%, a specificity of 57.5%, a PPV of 29.2%, and a NPV of 85.2%. The selected features are listed in **Supplementary Table S3**, and the corresponding ROC curves are shown in **Supplementary Figure S1**.

## 4 Discussion

In this study, the kinetic and radiomic features of tumors demonstrated moderate accuracy (AUC: 0.705 and 0.689 in the test and validation sets, respectively) to differentiate ALNM burden (≤2 or >2 nodes) in T1 and T2 stage breast cancer. However, the kinetic and radiomic features of the tumors exhibited a high NPV (92.1% or 90.2% in the test dataset and validation dataset, respectively), demonstrating their potential as a reliable rule-out test. The high NPV particularly supported their utility as a non-invasive tool to identify candidates for less invasive axillary procedures.

Our result was similar to that reported by Han Lu et al. (7), who demonstrated that a radiomic signature on MRI could moderately predict ALNM burden (≤2 or >2 nodes) (AUC of 0.79). However, our model’s performance was lower than that in another study (13), which reported an AUC of 0.88 for differentiating patients with one to two metastatic nodes from those with ≥3 metastatic nodes. A key methodological difference in our study was the incorporation of quantitative kinetic features alongside radiomic features. However, this addition did not enhance the overall predictive performance in our study. This result was different from that of another study (8), which found that combining kinetic and radiomic features yielded the highest performance for predicting the presence of ALNM (AUC of 0.91), outperforming the use of radiomic features alone. The discrepancy in conclusions may be attributed to a difference in endpoints: their combined model predicted ALNM status (positive/negative), whereas ours aimed to stratify by specific nodal burden (≤2 or >2 nodes).

Furthermore, as noted in the Introduction, studies using kinetic parameters to predict ALNM in breast cancer have yielded conflicting results (17–24). Our previous study (24) showed that quantitative kinetic parameters of the tumors with histogram and texture features were not associated with ALNM in 94 patients. The current study, despite its larger sample size and five kinetic features to be included in the model, still demonstrated moderate predictive performance. The distinct differences in the tumor microenvironment between the ALN and primary tumor site may account for this discrepancy, as suggested by prior research (10). Therefore, future efforts to improve MRI-based diagnostic accuracy for ALNM may benefit from focusing on kinetic or radiomic features derived directly from the ALNs themselves.

Indeed, several studies (26–28) using radiomic and kinetic features of ALNs have shown relatively high diagnostic performance for ALNM, with AUC values of 0.88, 0.86, and 0.979, respectively. Two previous studies (10, 12) also found that the radiomic signature of the ALN on MRI achieved the best performance in predicting SLN or ALN status, with AUC values of 0.906 and 0.85, respectively. Moreover, their analysis revealed that the ALN-derived radiomic signature outperformed both the combined (tumor+ALN) signature and the tumor signature alone in the test or validation cohorts. A fundamental obstacle in using ALN-derived features stems from the clinical dilemma of node-to-node matching. Although previous studies have demonstrated promising results, their inherent selection bias cannot be overlooked. For instance, to define positive lymph nodes, they applied a dual criterion requiring both a high metastatic burden (n > 8) confirmed by ALND and the presence of up to three highly suspicious lymph nodes on MRI (26, 27). Consequently, while these findings are encouraging, their broad generalizability remains uncertain. That was why we based our study on tumor features, rather than ALN characteristics, to predict ALNM burden.

Compared with previous studies that relied on tumor radiomics to differentiate metastatic ALNs from non-metastatic ALNs (not ALNM burden), our model demonstrated a comparable AUC to a few of them (7, 10, 12, 16). However, the model demonstrated comparatively lower performance (in terms of AUC, sensitivity, and specificity) than those reported in other studies (8, 9, 11, 13–15). This discrepancy may be attributed to several factors. 1) Feature extraction platform: our study relied on the Omni-Kinetics software, primarily for extracting kinetic features, which resulted in a relatively limited set of radiomic features. In contrast, comprehensive tools such as PyRadiomics or RadCloud are recommended in the field (29). 2) Feature selection method: the majority of studies utilized the least absolute shrinkage selection operator (LASSO) regression for feature selection, while our approach employed ANOVA with stepwise regression. Although this method was interpretable and allowed us to identify a parsimonious set of features, we acknowledged that more contemporary techniques like LASSO regression may offer advantages in handling multicollinearity. 3) Model building: numerous studies have documented improved AUC when clinical or pathological parameters were incorporated with radiomic data (9, 11, 13, 15). Our study focused on developing an imaging-based model and thus did not incorporate clinical parameters. 4) Cohort characteristics: unlike most prior studies, our cohort design —which was confined to T1 and T2 stage breast cancer and stratified by ALNM burden (≤2 or >2 nodes), resulted in an imbalanced dataset (for more details, see **Supplementary Table S4**). All the above factors also represented barriers to the clinical adoption of radiomics—including segmentation challenges, limited generalizability, and poor reproducibility (30).

This study had certain limitations. First, the manual segmentation may decrease reproducibility. Future studies could benefit from adopting automated detection and segmentation methods. Including spiculations within the segmentation may reduce feature reproducibility and distort radiomic features; however, research by Cama I. et al. has found that segmentation accuracy does not diminish their predictive capability (31). Second, our feature selection employed ANOVA with stepwise regression. We acknowledged that more contemporary techniques like LASSO regression may offer advantages in handling multicollinearity. However, the use of an independent test set for validation provides confidence that our model’s performance is generalizable. Third, the study was restricted to kinetic and radiomic features from MRI. The inclusion of clinical and pathological features (e.g., Ki-67 data) may improve the predictive performance. Fourth, the inclusion of pure DCIS cases may influence model performance, particularly by inflating the NPV. We included them to address a common preoperative dilemma that a core needle biopsy cannot definitively rule out concurrent invasive carcinoma. Given this diagnostic uncertainty, patients with an initial DCIS diagnosis thus represent a clinically relevant population requiring preoperative nodal assessment. Fifth, a high SLNB rate was observed in the group with ALNM ≤ 2 nodes, and its false-negative rate may have introduced potential bias into the results. Finally, this was a retrospective single-center investigation with a limited sample size. Thus, future research involving larger, multi-center cohorts is warranted to validate and extend our findings.

## 5 Conclusion

The kinetic and radiomic features from DCE-MRI showed potential for predicting ALNM burden (≤2 or >2 nodes) in T1 and T2 stage breast cancer. The high NPV particularly supported their utility as a non-invasive tool to identify candidates for less invasive axillary procedures.

## Data Availability

The raw data supporting the conclusions of this article will be made available by the authors, without undue reservation.
