# Supplementary material for: Kinetic and radiomic features on DCE-MRI as a predictor for axillary lymph node metastasis burden in T1 and T2 stage breast cancer
Source: Front Oncol. 2026 Jan 7;15:1700248. doi: 10.3389/fonc.2025.1700248 (PMC12819264; doi:10.3389/fonc.2025.1700248)
Supplement: Supplementary file 1 [file DataSheet1.docx]

**Supplementary Material:**

**Table S1** MRI protocol used in the study.

| MRI protocol | TR (ms) | TE (ms) | FOV (mm) | Slice Thickness (mm) | Matrix | Total Acquisition Time |
| --- | --- | --- | --- | --- | --- | --- |
| Pre-contrast enhanced sequences |  |  |  |  |  |  |
| Axial T2WI | 3570 | 70 | 340 × 340 | 4 | 358 × 448 | 3 min 29 s |
| Sagittal T2WI | 3600 | 56 | 200 × 200 | 3 | 358 × 448 | 2 min 47 s |
| T1 mapping | 5.64 | 2.46/3.69 | 360 × 360 | 2.5 | 269 × 384 | 58 s |
| Post-contrast enhanced sequence |  |  |  |  |  |  |
| CAIPIRINHA-Dixon-Twist-Vibe | 5.64 | 2.46 | 360 × 360 | 2.5 | 269 × 384 | 5 min 12 s |

TR, repetition time; TE, echo time; FOV, field of view; T2WI, T2-weighted imaging

**Table S2** Clinical and pathological characteristics after excluding DCIS cases.

| Variables | ALNM ≤ 2 nodes (n = 135) | ALNM > 2 nodes (n = 36) | *p*-Value |
| --- | --- | --- | --- |
| Age (years), median (IQR) | 47 (42, 56) | 48 (43,53) | 0.76 |
| Menopause (n，%) |  |  | 1.000 |
| Yes | 62 (46.0) | 16 (44.4) |  |
| No | 73 (54.0) | 20 (55.6) |  |
| Tumor size (mm), median (IQR) | 20 (16, 25) | 23 (18, 28) | 0.106 |
| Tumor stage (n，%) |  |  | 0.003 |
| T1 | 55 (40.7) | 5 (13.9) |  |
| T2 | 80 (59.3) | 31 (86.1) |  |
| Number of ALNM (n，%) |  |  | ＜ 0.001 |
| 0 | 89 (65.9) | 0 |  |
| 1-2 | 46 (34.1) | 0 |  |
| > 2 | 0 | 36 (100) |  |
| Axillary management (n，%) |  |  | ＜ 0.001 |
| SLNB | 95 (70.4) | 0 |  |
| ALND/SLNB+ALND | 40 (29.6) | 36 (100) |  |
| Histopathological type (n，%) |  |  | 0.627 |
| NST | 129 (95.6) | 35 (97.2) |  |
| ILC | 2 (1.5) | 1 (2.8) |  |
| Other types | 4 (2.9) | 0 |  |
| Pathological parameters (n，%) |  |  |  |
| ER positivity ^a^ | 111/135 (82.2) | 26/36 (72.2) | 0.238 |
| PR positivity ^a^ | 99/135 (73.3) | 21/36 (58.3) | 0.101 |
| HER2 positivity ^b^ | 38/135 (28.1) | 10/36 (27.8) | 0.453 |
| Ki-67 positivity ^c^ | 96/135 (71.1) | 33/36 (91.7) | 0.009 |
| Molecular subtype (n，%) |  |  | 0.086 |
| Luminal A | 30 (22.2) | 2 (5.6) |  |
| Luminal B | 81 (60.0) | 24 (66.7) |  |
| HER2-enrich | 10 (7.4) | 4 (11.1) |  |
| TNBC | 14 (10.4) | 6 (16.7) |  |

DCIS, ductal carcinoma *in situ*; ALNM, axillary lymph node metastasis; SLNB, sentinel lymph node biopsy; ALND axillary lymph node dissection; NST, no special type; ILC, invasive lobular carcinoma; ER, estrogen receptor; PR, progesterone receptor; HER2, human epidermal growth factor receptor 2; TNBC, triple-negative breast cancer; IQR, interquartile range.

^a^ER and PR positivity was defined as defined as ≥ 1% of tumor cells appearing immunostained

^b^HER2 positivity was defined as hematoxylin–eosin (H&E) staining 3+ or H&E staining 2+ with positive fluorescence in *in situ* hybridization test.

^c^Ki-67 positivity was considered as ≥ 14% cells appearing immunostained.

**Table S3** The selected features and their coefficients in the model after excluding DCIS cases.

| The selected kinetic and radiomic features | Coefficient in model |
| --- | --- |
| Min MAX Slope | 0.176 |
| Min TTP | -0.520 |
| Std TTP | 0.394 |
| AngularSecondMoment | 0.077 |
| LowGreyLevelRunEmphasis | -1.131 |
| sumEntropy | 0.422 |

DCIS, ductal carcinoma *in situ*; TTP, time to peak.


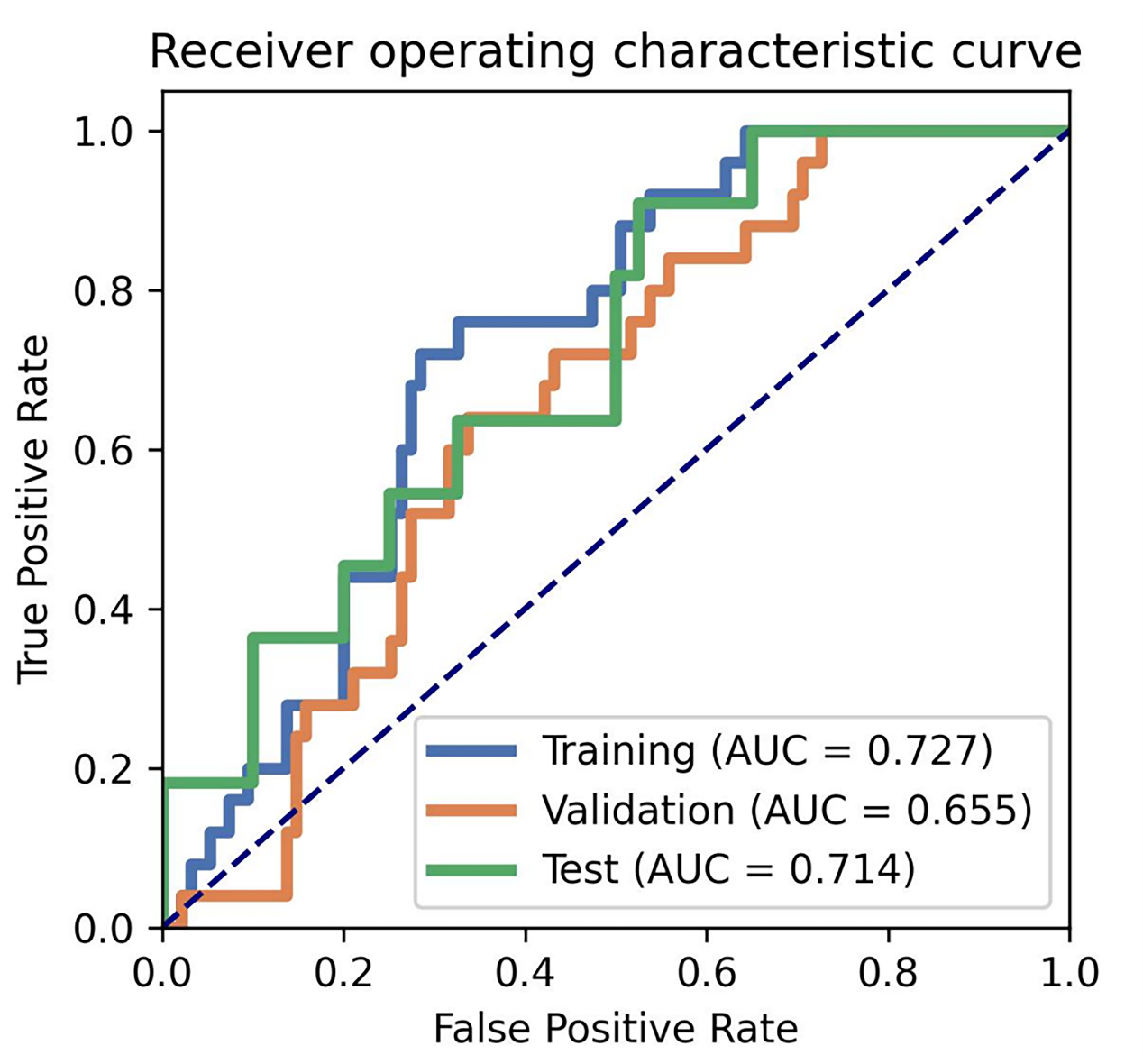


**Figure S1.** Receiver operating characteristic (ROC) curves of kinetic and radiomic feature model after excluding DCIS cases. DCIS, ductal carcinoma *in situ.*

**Table S4** Characteristics of studies using tumor radiomic features to predict SLN metastases or ALNM.

| Study | Study design type | Enrolled patient (n) | T stage | SLN or ALN  metastases | Patients with metastatic nodes vs. those with non-metastatic nodes (n) | Field of MRI | Tumor  segmentation | Feature extraction | Feature reduction and selection | Model achieving highest AUC in validation or test set | AUC (in validation or test cohort) |
| --- | --- | --- | --- | --- | --- | --- | --- | --- | --- | --- | --- |
| Han L  2019  [7] | Retro | 411 | ND | ALNM and ALNM burden | 148 vs. 263 | 1.5T | Manually, ITK-SNAP | PyRadiomics, 808 features | Mann-Whitney U test, LASSO, and LOOCV | Clinical-radiomic combined nomogram, and radiomic signature to distinguish the ALNM burden (≤ 2 or >2 nodes) | 0.87 and 0.79, respectively |
| Chen WY  2024  [13] | Retro | 410 | cT1-2 | ALNM and ALNM burden | 139 vs. 271 | 1.5T | Manually, 3D Slicer | Radcloud platform, 1409 features | Variance threshold methods, SelectKBest method, and LASSO | Clinical-radiomic combined model to distinguish N0 from N+, and to distinguish N+(1-2) from N+(≥3) | 0.859 and 0.881, respectively |
| Chai R  2019  [8] | Retro | 120 | ND | ALNM | 59 vs. 61 | 3.0T | Manually, Mazda | Mazda, 475 radiomic features from each of the four sequences (T1WI, T2WI, DWI and CE2), 109 kinetic features from DCE sequence | LASSO | CE2 radiomic features, and CE2 radiomic features and kinetic features | 0.85 and  0.9132,  respectively |
| Yu Y  2020  [10] | Retro | 1214 (multi-center) | T1-4 | ALNM | 544 vs. 670 | 1.5T or 3.0T | Semiautomatically, 3D Slicer | In-house texture extraction platform based on the Python package PyRadiomics, 2589 features from three sequences (T1+C, T2WI and DWI) | LASSO and random forest algorithm | Combined radiomic signature from different sequences of tumor, and combined radiomic signature of ALN and combination of tumor and ALN, respectively | 0.60,  0.85 and 0.71, respectively |
| Ma M  2022  [12] | Retro | 142 | T1-4 | SLN metastases | 52 vs. 90 | 3.0T | Automatically, 3D U-Net segmentation model in Python | PyRadiomics, 1070 radiomic features | recursive feature elimination, ANOVA, Kruskal–Wallis test, and relief | Radiomic signature of tumor, and radiomic signature of ALN and combination of tumor and ALN, respectively | 0.699, 0.906 and 0.696,  respectively |
| Wang Q  2024  [14] | Retro | 485 | cT1-3 | ALNM | 213 vs. 272 | 1.5T or 3.0T | Manually, 3D Slicer | PyRadiomics,1223 features of tumor, and 1223 features of ALN | Mann-Whitney U test, LASSO, and Spearman analysis | Radiomic signature of tumor and ALN in external validation cohort | 0.81 and 0.756, respectively |
| Tan H  2020  [9] | Retro | 329 | ND | ALNM | 119 vs. 210 | 3.0T | Manually, ITK-SNAP | In-house software implemented in MatLab 2019b, 647 features from T2WI | Mann-Whitney U test, and SVM | Clinical-radiomic combined nomogram | 0.81 |
| Our previous study  2021  [11] | Retro | 186 | ND | SLN metastases | 93 vs. 93 | 3.0T | Manually, Omni-Kinetics | Omni-Kinetics, 121 kinetic parameters and 77 radiomic features | mRMR, LASSO and the backward stepwise logistic regression | Radiomic signature of tumor, and nomogram for combining radiomics score of tumor and FGT with one clinical feature | 0.783 and 0.839, respectively |
| Li X  2025  [15] | Retro | 379  (multi-center) | T1-4 | ALNM | 186 vs. 193 | 1.5T or 3.0T | Manually,  ITK-SNAP | Pyradiomics, 1888 features | Mann-Whitney U test and correlation analysis, and the elastic network algorithm | Radiomic model, and clinical- radiomic combined nomogram in external validation cohort | 0.760 and 0.818, respectively |
| Zhang D  2025  [16] | Retro | 183 | ND | ALNM | 107 vs. 76 | 3.0T | 3D Slicer | Pyradiomics, 1138 and 923 features from DCE and DWI sequences | SelectKBest and LASSO | DCE model, and combined model (features from DCE and DWI sequences) | 0.760 and 0.859, respectively |
| Our study | Retro | 185 | T1-2, and Tis | ALNM burden | 36 vs. 149  (ALNM >2 vs. ≤2 nodes) | 3.0T | Manually, Omni-Kinetics | Omni-Kinetics, 121 kinetic features and 77 radiomic features | ANOVA with stepwise regression | Kinetic and radiomic feature model to distinguish the ALNM burden (≤ 2 or > 2 nodes) | 0.705 |

SLN, sentinel lymph node; ALNM, axillary lymph node metastasis; ALN, axillary lymph node; MRI, magnetic resonance imaging; AUC, area under the curve; ND, no data; LASSO, the least absolute shrinkage selection operator; LOOCV, the leave-one-out cross-validation; T1WI, T1-weighted imaging; T2WI, T2-weighted imaging; DWI, diffusing weighted imaging; CE2, the second postcontrast phase of the DCE sequence; DCE, dynamic contrast enhancement; ANOVA, analysis of variance; mRMR, minimal redundancy maximum relevancy; FGT, fibroglandular tissue.
